# Supplementary material for: 1,2-13C2-Glucose Tracing Approach to Assess Metabolic Alterations of Human Monocytes under Neuroinflammatory Conditions
Source: Curr Issues Mol Biol. 2023 Jan 16;45(1):765–81. doi: 10.3390/cimb45010051 (PMC9857935; doi:10.3390/cimb45010051)
Supplement: Supplementary file 1 [file cimb-45-00051-s001.zip › cimb-2118434-supplementary.pdf]

Supplementary materials

Table S1. Concentrations of the targeted analytes in cell lysate and culture medium. The reported concentrations are mean (± SD) values of three biological replicates.

	Cell lysate				Incubation medium			
	**NS**	**CON**	**AD**	**MScl**	**NS**	**CON**	**AD**	**MScl**
	μg/mL±SD	μg/mL±SD	μg/mL±SD	μg/mL±SD	μg/mL±SD	μg/mL±SD	μg/mL±SD	μg/mL±SD
Pyruvate	1.30±0.60	2.48±0.09	2.35±0.44	1.87±0.45	3.49±0.12	8.21±0.65	8.38±0.70	7.39±0.98
2,3-$^{13}C_2$ pyruvate	0.55±0.19	0.78±0.08	0.710±0.14	0.623±0.19	1.43±0.09	2.25±0.13	2.32±0.05	2.16±0.19
Lactate	41.20±8.32	43.79±2.57	40.40±6.05	35.48±7.35	48.00±3.04	41.65±0.95	51.36±4.63	54.12±4.23
1,2-$^{13}C_2$ lactate	7.26±1.16	8.04±0.84	6.76±1.02	6.80±1.26	8.09±0.96	7.36±0.85	7.82±0.59	9.53±0.56
2-^{13}C glycine	n.q.	n.q.	n.q.	n.q.	0.15*	n.q.	n.q.	n.q.
Glycine	6.68±1.63	4.73±0.45	3.96±0.55	3.93±0.82	12.22±0.14	8.75±0.35	8.22±0.19	8.96±0.49
1,2-$^{13}C_2$ glutamine	n.q.	0.05±0.01	0.04±0.01	0.031*	n.q.	0.09±0.01	0.09±0.004	0.06±0.01
Glutamine	1.27±0.35	6.40±0.94	5.56±0.98	3.71±1.02	2.24±0.07	12.35±1.04	12.39±0.64	9.24±1.04
Serine	12.51±3.80	8.30±0.82	6.97±0.96	6.91±1.82	25.24±0.20	17.41±0.39	15.65±0.09	16.95±0.19
Glutamic acid	7.67±2.94	8.44±0.98	6.81±1.05	7.43±1.32	17.12±0.51	16.32±0.62	13.77±0.79	12.83±0.25
Citric acid	2.99±2.51	10.81±1.24	10.58±2.76	7.78±1.59	11.90±1.87	40.90±1.44	41.27±1.91	34.12±4.58

* Only one sample over the three biological replicates presented the analyte; n.q. denotes “not quantifiable”.
